# Nuclear translocation of beta-catenin in colorectal cancer

**DOI:** 10.1054/bjoc.1999.1112

**Published:** 2000-04-27

**Authors:** M Kobayashi, T Honma, Y Matsuda, Y Suzuki, R Narisawa, Y Ajioka, H Asakura

**Affiliations:** Third Department of Internal Medicine and First Department of Pathology, Niigata University School of Medicine, Asahimachi-dori-1-757, Niigata, 951-8122, Japan

**Keywords:** beta-catenin, immunohistochemistry, colorectal cancer, familial adenomatous polyposis (FAP)

## Abstract

Post-translational stabilization of beta-catenin through mutation of the adenomatous polyposis coli (APC) gene has been proposed as an early step in colorectal carcinogenesis. Beta-catenin may translocate from the cytoplasm to the nucleus, where it might serve as a transcriptional factor to stimulate tumour formation. We investigated intracellular localization of beta-catenin in sporadic colorectal adenomas and cancers as well as familial adenomatous polyposis (FAP). Nuclear over-expression of beta-catenin was observed in 35% (7/20) of intramucosal cancers and 42% (23/55) of invasive cancers but was not seen in any adenomas from sporadic or FAP cases. Cytoplasmic beta-catenin in adenomas was significantly higher than that of normal mucosa in both sporadic and FAP cases. The cytoplasmic intensity index of cancers was significantly higher than that of sporadic adenomas, but the index was not correlated with nuclear expression in cancers. These findings suggest that nuclear translocation of beta-catenin is involved in development of intramucosal cancer rather than adenoma, independent of APC mutations. Cytoplasmic accumulation of beta-catenin may occur in adenomas, but it remains to be determined whether this is a cause or a consequence of colorectal cancer. © 2000 Cancer Research Campaign


British Journal of Cancer (2000) 82(10), 1689–1693
© 2000 Cancer Research Campaign
DOI: 10.1054/ bjoc.1999.1112, available online at http://www.idealibrary.com on IDEAL®

### Nuclear translocation of beta-catenin in colorectal cancer

**M Kobayashi[1], T Honma[1], Y Matsuda[1], Y Suzuki[1], R Narisawa[1], Y Ajioka[2] and H Asakura[1]**

[1]Third Department of Internal Medicine and [2]First Department of Pathology, Niigata University School of Medicine, Asahimachi-dori-1-757, Niigata, 951-8122, Japan

**Summary** Post-translational stabilization of beta-catenin through mutation of the adenomatous polyposis coli (APC) gene has been proposed as an early step in colorectal carcinogenesis. Beta-catenin may translocate from the cytoplasm to the nucleus, where it might serve as a transcriptional factor to stimulate tumour formation. We investigated intracellular localization of beta-catenin in sporadic colorectal adenomas and cancers as well as familial adenomatous polyposis (FAP). Nuclear over-expression of beta-catenin was observed in 35% (7/20) of intramucosal cancers and 42% (23/55) of invasive cancers but was not seen in any adenomas from sporadic or FAP cases. Cytoplasmic beta-catenin in adenomas was significantly higher than that of normal mucosa in both sporadic and FAP cases. The cytoplasmic intensity index of cancers was significantly higher than that of sporadic adenomas, but the index was not correlated with nuclear expression in cancers. These findings suggest that nuclear translocation of beta-catenin is involved in development of intramucosal cancer rather than adenoma, independent of APC mutations. Cytoplasmic accumulation of beta-catenin may occur in adenomas, but it remains to be determined whether this is a cause or a consequence of colorectal cancer. © 2000 Cancer Research Campaign

**Keywords**: beta-catenin; immunohistochemistry; colorectal cancer; familial adenomatous polyposis (FAP)

The adenoma–carcinoma sequence of colorectal tumorigenesis requires accumulation of genetic alterations. Inactivation of the adenomatous polyposis coli (APC) suppressor gene is the first event in this sequence, which results in colorectal adenoma formation (Vogelstein et al, 1988). Recently, it has been reported that APC mutations are associated with an accumulation of intracellular beta-catenin protein, which leads to loss of control of normal beta-catenin signalling (Munemitsu et al, 1995). Thus, beta-catenin may promote colorectal adenoma formation resulting from the APC mutation. The beta-catenin protein is involved in two major functions: cell adhesion and mediation of the Wingless/Wnt signal transduction pathway (Behrens et al, 1996; Barth et al, 1997). Signal transduction of beta-catenin involves post-translational stabilization and its passage into the nucleus, where it interacts with transcription factors of the T-cell factor or lymphoid enhancer factor (LEF) family to activate target genes regulating cell proliferation and apoptosis (Korinek et al, 1997; Morin et al, 1997). Therefore, the specific localization of beta-catenin may influence its oncogenic activity. Beta-catenin has been reported in the nuclei and cytoplasm of adenomas and cancers from patients with familial adenomatous polyposis (FAP) but not in sporadic cancers (Inomata et al, 1996). Another study showed nuclear localization with increased expression in most sporadic adenomas (Valizadeh et al, 1997). To determine if intracellular localization of beta-catenin might be involved in carcinogenesis, we investigated beta-catenin localization in sporadic colorectal adenomas and cancers and in patients with FAP.

*Received 2 June 1999*
*Revised 2 November 1999*
*Accepted 20 December 1999*

*Correspondence to:* M Kobayashi

### MATERIALS AND METHODS

### Specimen preparation

Lesions ($n$ = 85) were resected endoscopically ($n$ = 53) or surgically ($n$ = 22) from 75 patients with sporadic colorectal adenomas and cancers. Tubular adenomas ($n$ = 29), intramucosal cancers ($n$ = 12) and invasive cancers ($n$ = 44) were evaluated. We also studied tubular adenomas ($n$ = 63), intramucosal cancers ($n$ = 8) and invasive cancers ($n$ = 11) obtained surgically from 12 patients with FAP. Six of the 12 FAP patients had adenomatous polyposis without cancer. The 75 cancers were well- ($n$ = 62), moderately- ($n$ = 11) and poorly-differentiated ($n$ = 2) adenocarcinomas. Specimens were fixed in 10% formaline and embedded in paraffin. Serial sections were prepared for haematoxylin and eosin (H&E) staining and immunostaining.

### Immunohistochemisty

The streptavidin-biotin immunoperoxidase method was employed for immunostaining of beta-catenin protein. Deparaffinized and rehydrated sections were heated in a microwave oven in 0.01 mol $l^{-1}$ sodium citrate buffer (pH 6.5) for seven 3-min cycles. Endogenous peroxidase activity was inhibited by incubation with 0.3% hydrogen peroxidase in methanol for 20 min. After blocking any nonspecific reactions with 10% normal rabbit serum, the sections were incubated with anti-beta-catenin antibody (1:250, Transduction Labs, Lexington, KY, USA) for 1 h. Sections were then incubated with biotinylated rabbit anti-mouse immunoglobulin for 30 min followed by exposure to streptavidin–peroxidase complex (Histofine SAB-PO kit, Nichirei, Tokyo, Japan) for 30 min. Colour was then developed with diaminobenzidine solution. Sections were then lightly counterstained with haematoxylin and mounted.

### Evaluation of beta-catenin immunostaining

Nuclear staining of positive cells was defined as intense brown colour in the nucleus. The pattern of nuclear immunostaining was classed as follows (Figure 1): negative or scattered group, no or very few scattered positive cells without any clusters; focal group, positive cells clustered in focal areas; diffuse, over-expressed group, positive cells distributed diffusely, homogeneously or heterogeneously.

For evaluation of immunostaining intensity in the cytoplasm, microscopic images of immunostained sections were measured for optical density using an image analysis system (SP-500, Olympus, Tokyo, Japan). In each section, the areas selected were equivalent to a 100 × total magnification lens field. Cytoplasmic intensity of tumours (T) was analysed by randomly processing 100 tumour cells per section at a magnification of 400 ×. As an internal control for each section, normal cytoplasmic intensity (N) was measured in 100 normal mucosa cells near the mucosal surface; immunoreactivity of mucosal cells increases gradually from the base of the crypts to the luminal surface (Inomata et al, 1996). We defined the cytoplasmic intensity index as follows: (T−N)/mean N of each group.

Staining intensity of the cell membrane was compared with normal mucosa cells as an internal positive control; these were divided into two groups. Tumour cells found to be homogeneously positive were described as a preserved type, while cases with weak staining were described as a reduced type.

We correlated subcellular immunoreactivity with histopathological findings, size of adenoma and invasion of cancer. The

**B**

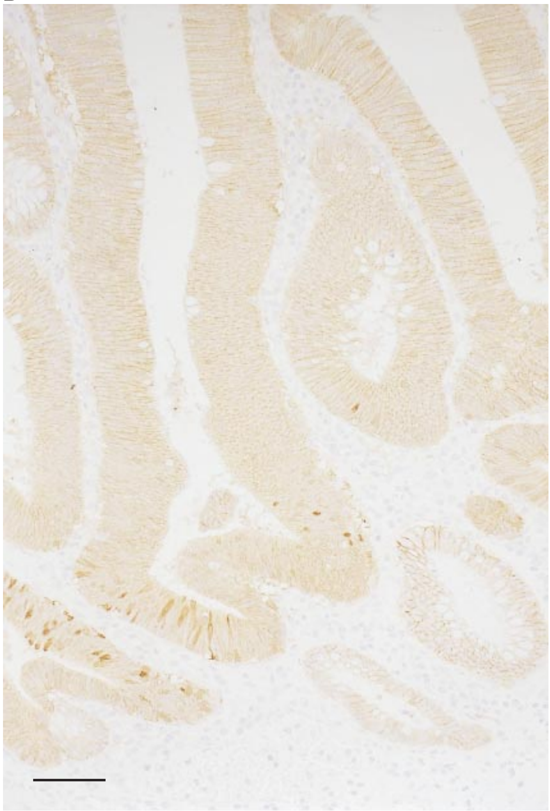

**A**

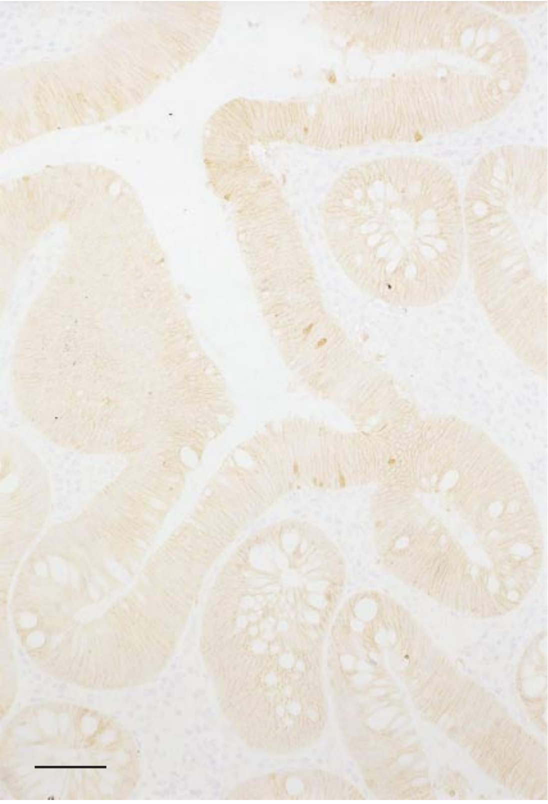

**C**

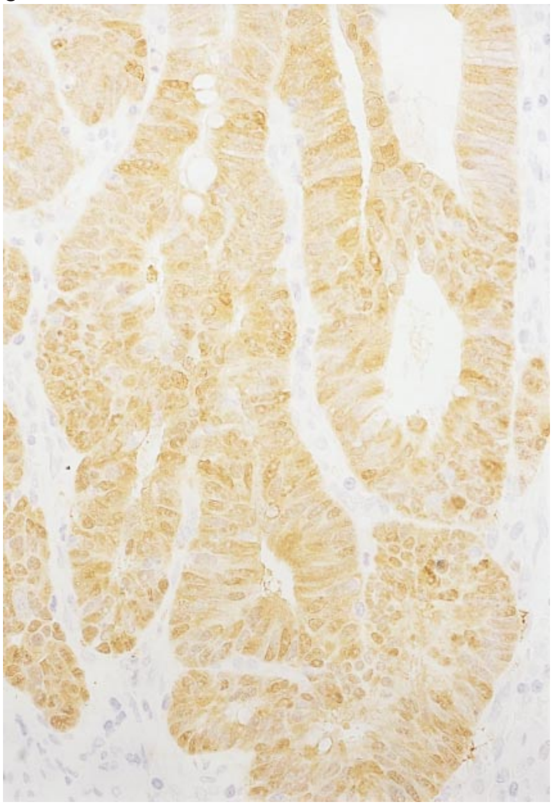

**Figure 1** Three patterns of nuclear immunostaining of beta-catenin. (**A**) Negative/scattered group, few (or no) nuclear-positive cells scattered without clusters; bar = 100 μm. (**B**) Focal group, nuclear-positive cells clustered in focal areas; bar = 100 μm. (**C**) Diffuse, over-expressed group, nuclear-positive cells distributed diffusely, homogeneously or heterogeneously; bar = 50 μm

*© 2000 Cancer Research Campaign*

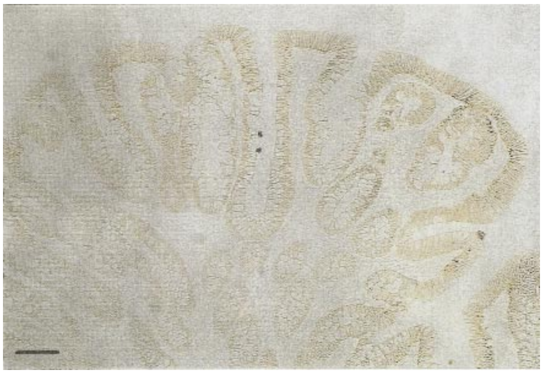

**Figure 2** Immunohistochemical staining of beta-catenin in a tubular adenoma (1 mm) from a patient with familial adenomatous polyposis. Cell membrane immunostaining is preserved without expression in the nucleus; bar = 100 μm cytoplasmic intensity of the adenoma or cancer was compared to normal mucosa using a paired *t*-test. The cytoplasmic intensity index was compared using the nonparametric Mann–Whitney *U*-test and the Kruskal–Wallis test. The association of nuclear expression with cell membrane expression was examined by the $\chi^2$ test. Probability values smaller than 0.05 were considered statistically significant.

### RESULTS

### Nuclear expression of beta-catenin

Nuclear immunoreactivity and the histopathology of colorectal tumours are presented in Table 1. Diffuse positivity was not detected in any adenomas from sporadic or FAP (Figure 2) cases. There was no correlation between nuclear expression and histological differentiation of cancers. The nuclear positivity of 30 tumours in which cancerous and adenomatous areas were co-existing is shown in Table 2. Nine cancers with simultaneous adenomas showed diffuse immunostaining in cancer areas. However, eight of the nine tumours did not show diffuse nuclear staining in adenoma components (Figure 3).

### Cytoplasmic expression of beta-catenin

Cytoplasmic staining intensity was significantly ($P < 0.001$) higher in adenomas (112.2 ± 15.7, mean ± s.d.) and cancers (116.2 ± 18.8) than in normal mucosa (96.7 ± 12.3), in both sporadic and FAP cases. The cytoplasmic staining intensity index and histopathology are presented in Figure 4. The cytoplasmic intensity index of invasive cancers was significantly higher than that of adenomas in both sporadic and FAP cases. There was no correlation between cytoplasmic intensity index and nuclear positivity in either sporadic or FAP cancers.

### Cell membrane expression of beta-catenin

All adenomas from both sporadic and FAP cases showed the preserved type of cell membrane immunostaining. The reduced type was significantly ($P < 0.02$) associated with nuclear expression in cancers from both sporadic and FAP cases (Table 3). There was no correlation between cell membrane immunostaining pattern and cytoplasmic intensity index.

**Table 1** Relationship between nuclear expression of beta-catenin and the histopathology of colorectal adenomas and cancers

| Histopathology | Negative/scattered | Focal | Diffuse |
|---|---|---|---|
| Sporadic adenoma | | | |
| < 5 mm | 13 (100)[a] | 0 (0) | 0 (0) |
| ≥ 5 mm | 14 (88) | 2 (13) | 0 (0) |
| Sporadic cancer | | | |
| Intramucosal | 5 (42) | 3 (25) | 4 (33) |
| Invasive | 24 (55) | 3 (7) | 17 (39) |
| FAP adenoma | | | |
| < 5 mm | 43 (100) | 0 (0) | 0 (0) |
| ≥ 5 mm | 20 (100) | 0 (0) | 0 (0) |
| FAP cancer | | | |
| Intramucosal | 1 (13) | 4 (50) | 3 (38) |
| Invasive | 3 (27) | 2 (18) | 6 (55) |

[a]Numbers in parentheses are percentages. Negative/scattered, no or few scattered positive cells; Focal, positive cells clustered in focal areas; Diffuse, positive cells distributed diffusely. FAP, familial adenomatous polyposis.

### DISCUSSION

In this study, nuclear over-expression of beta-catenin was observed in 35% (7/20) of intramucosal cancers and 42% (23/55) of invasive cancers but was not observed in adenomas from either sporadic or FAP cases. The frequency of invasive cancers with nuclear over-expression was higher than the 20% (32/154) value of a previous report (Günther et al, 1998). We observed that most cancers which were accompanied by adenomas showed nuclear over-expression of beta-catenin in the cancer area, but this was not seen in the adenoma component. Although we did not examine APC mutation, inherited mutations of APC cause FAP, and acquired APC mutations are present in approximately 80% of sporadic colorectal adenomas and cancers (Miyoshi et al, 1992; Jen et al, 1994). Thus, our findings suggest that nuclear translocation of beta-catenin is involved in the initiation of intramucosal cancers rather than adenomas, independent of APC mutations. The discrepancy with a previous study (Valizadeh et al, 1997) which showed intense nuclear staining in adenomas may be explained by different evaluations of immunostaining. Valizadeh et al (1997) did not examine the frequency or distribution of nuclear-positive cells. We distinguished the over-expressed staining pattern with positive cells distributed diffusely and frequently from the pattern of a few scattered positive cells or clustered positive cells in focal areas. We consider the over-expressed pattern significant for tumour formation. Another possible explanation for this discrepancy is different histological criteria for intramucosal cancers versus adenomas with severe to moderate dysplasia (Schlemper et al, 1998). Our results do not agree with the findings of Inomata et al (1996), who found that beta-catenin was localized predominantly to the nucleus of adenomas in FAP patients. Although they used a different fixation method, this discrepancy cannot be explained by specimen fixation in light of our results in cancers accompanied by adenomas. Inomata et al (1996) did not describe the number or size of the adenomas showing nuclear accumulation. We did not find over-expressed staining in any lesions of 43 FAP adenomas which were smaller than 5 mm (Figure 2). We suggest that nuclear accumulation may not be observed in early-stage adenomas in FAP patients.

© 2000 Cancer Research Campaign 

**Table 2** Nuclear expression of beta-catenin in colorectal cancers accompanied by adenomas

| | Adenoma area | | |
|---|---|---|---|
| Cancer area | Negative/scattered | Focal | Diffuse |
| Negative/scattered | 13 (100)[a] | 0 (0) | 0 (0) |
| Focal | 4 (50) | 4 (50) | 0 (0) |
| Diffuse | 7 (78) | 1 (11) | 1 (11) |

[a]Numbers in parentheses are percentages. Negative/scattered, no or few scattered positive cells; Focal, positive cells clustered in focal areas; Diffuse, positive cells distributed diffusely.

**Table 3** Relationship between cell membrane and nuclear expression of beta-catenin in colorectal cancers

| | Nuclear expression | | |
|---|---|---|---|
| Cell membrane expression | Negative/scattered | Focal | Diffuse |
| Preserved | 27 (61)[a] | 6 (14) | 11 (25) |
| Reduced | 6 (19) | 6 (19) | 19 (61) |

[a]Numbers in parentheses are percentages. Negative/scattered, no or few scattered positive cells; Focal, positive cells clustered in focal areas; Diffuse, positive cells distributed diffusely. Cell membrane expression was significantly associated with nuclear expression ($P < 0.02$ by $\chi^2$ test).

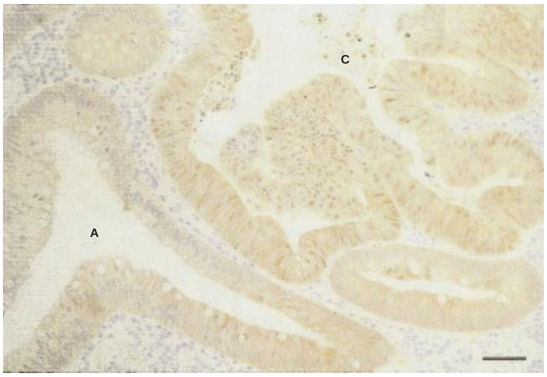

**Figure 3** Immunohistochemical staining of beta-catenin in a sporadic cancer accompanied by an adenoma (15 mm). There is diffuse nuclear staining in the cancer (**C**), but few positive cells in the adenoma (**A**); bar = 50 µm

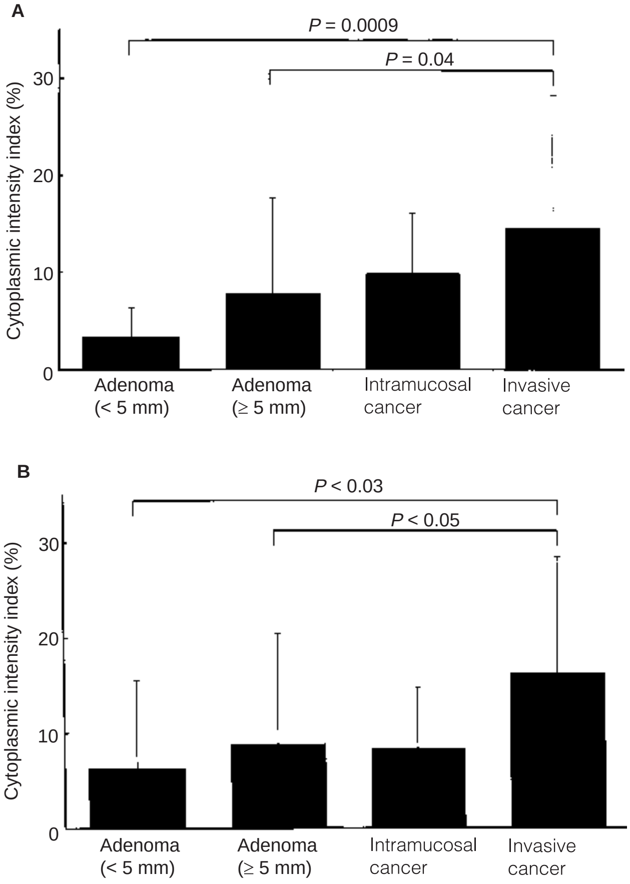

**Figure 4** The relationship between cytoplasmic intensity index of beta-catenin and the histopathology of colorectal adenomas and cancers from patients with sporadic (**A**) and familial adenomatous polyposis (**B**). Columns and bars represent the mean and standard deviation. For statistical analysis, the Mann–Whitney and Kruskal–Wallis tests were used

To investigate the cause of nuclear translocation of beta-catenin, we performed polymerase chain reaction (PCR)-single-strand conformational polymorphism (SSCP) and DNA sequence analysis for a mutation in exon 3 of the beta-catenin gene (CTNNB1), using 18 specimens of cancers with nuclear over-expression. It has been reported that mutations of CTNNB1 can increase free cytoplasmic beta-catenin (Morin et al, 1997). In only one of the 18 specimens, however, was a beta-catenin gene mutation found (serine site, data not shown). This result is compatible with previous reports that mutations in CTNNB1 are uncommon in colorectal cancers (Kitaeva et al, 1997; Günther et al, 1998; Iwao et al, 1998; Müller et al, 1998; Samowitz et al, 1999). Recently, in contrast to the Wnt signal transduction pathway, activated integrin-linked kinase (ILK) has been shown to induce translocation of beta-catenin from the plasma membrane to the nucleus, as well as induce the formation of the LEF-1/beta-catenin complex without an increase in the free pool of cytosolic beta-catenin (Novak et al, 1998). This evidence agrees with our finding that nuclear positivity is significantly associated with the reduced type of cell membrane staining in cancers. Further investigations are needed to reveal the mechanisms underlying nuclear translocation of beta-catenin.

Cytoplasmic staining intensity was significantly higher in adenomas than in normal mucosa, irrespective of adenoma size. Although cytoplasmic beta-catenin was frequently expressed at high levels in invasive cancers, it was not correlated with nuclear expression of beta-catenin. Stabilized beta-catenin should pass into the nucleus to interact with transcription factors and activate target genes (Korinek et al 1997; Morin et al, 1997). Therefore, it is likely that cytoplasmic accumulation of beta-catenin starts at the adenoma stage, but it is unknown whether cytoplasmic accumulation is a cause or a result of the progression from adenoma to cancer.

Inactivation of APC has been proposed as an initial step in adenoma formation. Our findings in adenomas of FAP patients suggest that APC inactivation is not sufficient to promote beta-catenin nuclear accumulation. It has been reported that the APC

 © 2000 Cancer Research Campaign gene regulates the cytoskeleton and promotes cell migration (Näthke et al, 1996). Loss of these APC functions might be involved in adenoma formation. P53 mutations are known to occur at an early stage of carcinoma development (Vogelstein et al, 1988), similar to nuclear localization of beta-catenin. However, we did not observe any correlation with p53 expression (data not shown).

In summary, we investigated the status of beta-catenin in the development of colorectal tumours. We confirmed that localization of beta-catenin, especially translocation to the nucleus, may be associated with progression through the adenoma–carcinoma sequence.

### ACKNOWLEDGEMENTS

This work was supported in part by a grant from the Sumitomo Foundation.

### REFERENCES

Barth AI, Näthke IS and Nelson WJ (1997) Cadherins, catenins and APC protein: interplay between cytoskeletal complexes and signaling pathways. *Curr Opin Cell Biol* **9**: 683–690

Behrens J, von Kries J, Kuhl M, Bruhn L, Wedlich D, Grosschedl R and Birchmeier W (1996) Functional interaction of β-catenin with the transcription factor LEF-1. *Nature* **382**: 638–642

Günther K, Brabletz T, Kraus C, Dworak O, Reymond MA, Jung A, Hohenberger W, Kirchner T, Köckerling F and Ballhausen WG (1998) Predictive value of nuclear beta-catenin expression for the occurrence of distant metastases in rectal cancer. *Dis Colon Rectum* **41**: 1256–1261

Inomata M, Ochiai A, Akimoto S, Kitano S and Hirohashi S (1996) Alteration of β-catenin expression in colonic epithelial cells of familial adenomatous polyposis patients. *Cancer Res* **56**: 2213–2217

Iwao K, Nakamori S, Kameyama M, Imaoka S, Kinoshita M, Fukui T, Ishiguro S, Nakamura Y and Miyoshi Y (1998) Activation of the β-catenin gene by interstitial deletions involving exon 3 in primary colorectal carcinomas without adenomatous polyposis coli mutations. *Cancer Res* **58**: 1021–1026

Jen J, Powell SM, Papadopoulos N, Smith KJ, Hamilton SR, Vogelstein B and Kinzler KW (1994) Molecular determinants of dysplasia in colorectal lesions. *Cancer Res* **54**: 5523–5526

Kitaeva MN, Grogan L, Williams JP, Dimond E, Nakahara K, Hausner P, DeNobile JW, Soballe PW and Kirsch IR (1997) Mutations in β-catenin are uncommon in colorectal cancer occurring in occasional replication error-positive tumors. *Cancer Res* **57**: 4478–4481

Korinek V, Barker N, Morin PJ, van Wichen D, de Weger R, Kinzler KW, Vogelstein B and Clevers H (1997) Constitutive transcriptional activation by a β-catenin-Tcf complex in APC−/− colon carcinoma. *Science* **275**: 1784–1787

Miyoshi Y, Nagase H, Ando H, Horii A, Ichii S, Nakatsuru S, Aoki T, Miki Y, Mori T and Nakamura Y (1992) Somatic mutations of the APC gene in colorectal tumors: mutation cluster region in the APC gene. *Hum Mol Genet* **1**: 229–233

Morin PJ, Sparks AB, Korinek V, Barker N, Clevers H, Vogelstein B and Kinzler KW (1997) Activation of β-catenin-Tcf signaling in colon cancer by mutations in β-catenin or APC. *Science* **275**: 1787–1790

Müller O, Nimmrich I, Finke U, Friedl W and Hoffmann I (1998) A β-catenin mutation in a sporadic colorectal tumor of the RER phenotype and absence of β-catenin germline mutations in FAP patients. *Genes Chromosomes Cancer* **22**: 37–41

Munemitsu S, Albert I, Souza B, Rubinfeld B and Polakis P (1995) Regulation of intracellular β-catenin levels by the adenomatous polyposis coli (APC) tumor-suppressor protein. *Proc Natl Acad Sci USA* **92**: 3046–3050

Näthke IS, Adams CL, Polakis P, Sellin JH and Nelson WJ (1996) The adenomatous polyposis coli tumor suppressor protein localizes to plasma membrane sites involved in active cell migration. *J Cell Biol* **134**: 165–179

Novak A, Hsu SC, Leung-Hagesteijn C, Radeva G, Papkoff J, Montesano R, Roskelley C, Grosschedl R and Dedhar S (1998) Cell adhesion and the integrin-linked kinase regulate the LEF-1 and β-catenin signaling pathways. *Proc Natl Acad Sci USA* **95**: 4374–4379

Samowitz WS, Powers MD, Spirio LN, Nollet F, van Roy F and Slattery ML (1999) β-catenin mutations are more frequent in small colorectal adenomas than in larger adenomas and invasive carcinomas. *Cancer Res* **59**: 1442–1444

Schlemper RJ, Itabashi M, Kato Y, Lewin KJ, Ridell RH, Shimoda T, Sipponen P, Stolte M and Watanabe H (1998) Differences in the diagnostic criteria used by Japanese and Western pathologists to diagnose colorectal carcinoma. *Cancer* **82**: 60–69

Valizadeh A, Karayiannakis AJ, El-Hariry I, Kmiot W and Pignatelli M (1997) Expression of E-cadherin-associated molecules (α, β, and γ-catenin and p120) in colorectal polyps. *Am J Pathol* **150**: 1977–1984

Vogelstein B, Fearon ER, Hamilton SR, Kerm SE, Preisinger AC, Leppert M, Nakamura Y, White R, Smitts Amm and Bos JL (1988) Genetic alterations during colorectal-tumour development. *N Engl J Med* **319**: 525–532

*© 2000 Cancer Research Campaign*
